# Valorization of Avocado Leaf as a By‐Product of the Avocado Fruit: Nutritional, Textural, and Sensory Properties of Gluten‐Free Erişte (Turkish Noodles)

**DOI:** 10.1002/fsn3.70615

**Published:** 2025-07-14

**Authors:** Başak Öncel, Nesibe Eryilmaz

**Affiliations:** ^1^ Department of Hotel, Restaurant and Catering Services University of Toros Mersin Turkey; ^2^ Department of Nutrition and Dietetics University of Toros Mersin Turkey

**Keywords:** antioxidant capacity, avocado leaf powder, functional ingredients, gluten‐free Erişte, sensory analysis

## Abstract

This study investigated the potential use of avocado leaf powder (ALP), a food waste product with high phenolic content and antioxidant capacity, in producing gluten‐free Erişte (GFE). For this purpose, GFE samples were formulated by replacing rice flour with different levels of ALP (5%, 10%, and 15%) and evaluated in terms of nutritional, physicochemical, textural, and sensory properties. Increasing the ALP content in the formulations led to gradual increases in ash (0.65%–1.33%), protein (11.35%–16.04%), fat (4.09%–4.16%), total dietary fiber (1.03%–7.13%), total phenolic content (46.74–90.63 mg GAE/100 g), antioxidant capacity (27.78–50.44 mg Trolox/100 g), mineral content, hardness, chewiness, and resistant starch (2.81%–6.26%). Conversely, cooking loss (10.53%–6.47%), adhesiveness, total starch content (74.59%–65.17%), and glycemic index (82.22%–65.85%) decreased with higher ALP incorporation. The cooking process increased L* in all samples while reducing a* and b* color parameters. According to sensory analysis results, GFE15 received the highest scores for color, aroma, hardness, and stickiness, whereas GFE10 had the highest overall acceptability score. Avocado leaves have been successfully applied as a functional ingredient in enriching gluten‐free noodles, demonstrating their suitability for functional noodle production and high nutritional value.

## Graphical abstract

This study explores the incorporation of ALP, a food waste by‐product, into gluten‐free extrudate (GFE) formulations and evaluates its effects on nutritional, textural, and sensory properties. ALP enrichment significantly enhanced protein, fiber, phenolic content, and antioxidant activity while reducing glycemic index. The GFE10 sample showed the highest overall sensory acceptance, highlighting ALP's potential as a functional ingredient in gluten‐free food development.

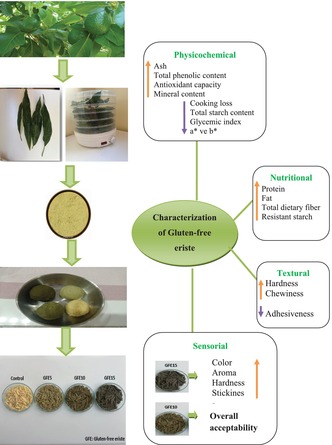

## 1 Introduction

Erişte (Turkish Noodle) is a food product that can be consumed by kneading the dough prepared with wheat flour, salt, alkaline salts (sodium carbonate, potassium carbonate, and sodium phosphate), eggs, and water, drying, and cooking with various methods (Kemahlıoğlu and Demirağ 2018). Recently, interest in Erişte consumption has increased due to its easy preparation–cooking process, high nutritional value, low cost, and good organoleptic properties (Ainsa et al. 2021). However, Erişte is not consumed by some consumers due to Celiac disease, which is a global disease and occurs as a result of the consumption of foods containing gluten (wheat, barley, rye) (Bouasla et al. 2017). GFE formulations for the consumption of celiac patients are being developed, and new products are on the market shelves. However, using corn, rice flour/starch as a wheat substitute in products leads to noteworthy changes in macro (sugar, lipid)–micronutrients, textural, and sensory (color, taste, appearance) properties (Fradinho et al. 2019). Moreover, gluten‐free products tend to have higher glycemic index values due to the raw materials used, which can negatively affect consumer health (Restuccia et al. 2023). In order to solve the mentioned technological and sensory problems and improve the nutritional value, gluten‐free noodle formulation has been enriched with wheat germ (Demir et al. 2021), quinoa flour (Çalışkan Koç and Pandiselvam 2022), grape, pomegranate, and rosehip seed flours (Koca et al. 2018), and mushroom flour (Süfer 2022). Recently, interest in gluten‐free nutrition has affected many people, except celiac patients, with the influence of environmental awareness and popular culture (Cardo et al. 2021). This situation reveals the need to develop new products in the gluten‐free market and offer them to consumers. However, increasing population and decreasing food resources may hinder these product development efforts. Solving the problem of food waste and using waste in food formulations can be an effective strategy to overcome these challenges (Tesfaye et al. 2022). In particular, avocado leaves, rich in phytochemical compounds and considered agricultural waste, have significant potential (Jimenez et al. 2021). Originating from Central America, avocados are cultivated in many parts of the world (Mexico, Dominican Republic, Peru, Colombia, Brazil, Brazil, Indonesia, and Turkey) due to their intriguing nutritional composition and are vital in the world food market (Yamassaki et al. 2017).

Avocado is considered a superfood due to its high phytochemical content (polyphenols, carotenoids, tocopherols, and sterols) (Bhuyan et al. 2019). Leaf, seed, and fruit peel, which are by‐products of avocado and used as waste, are also very rich in bioactive components (Yamassaki et al. 2017). Among these, avocado leaves stand out due to their high nutritional value (25%–28% protein, 38%–43% fiber, 7%–8% carbohydrates) and bioactive components, including flavonoids (epicatechin, luteolin, rutin) and carotenoids (α‐carotene, β‐carotene, lutein), as well as their potent antioxidant properties (Arukwe et al. 2012; Gümüştepe et al. 2022). Additionally, avocado leaves have the potential to lower the glycemic index, contributing to better blood sugar management and offering health benefits, particularly in gluten‐free product formulations (Arukwe et al. 2012; Gümüştepe et al. 2022; Rahman 2023).

When the literature was examined, studies on avocado leaves were found; however, no research has investigated their use as an enrichment agent in product formulations. Therefore, this study focused on producing GFE (Turkish noodles) enriched with 5%, 10%, and 15% ALP and examined its physical, chemical, nutritional, textural, and sensory properties. The primary objectives were to evaluate the applicability of ALP as an enrichment agent in GFE formulation, assess its impact on product characteristics, and develop a novel gluten‐free food product utilizing ALP as an agricultural by‐product.

## 2 Materials and Methods

### 2.1 Material

Avocado leaves were collected from Yenişehir, Mersin, Turkey (36.79154°N, 34.59494°E). After harvesting, the leaves were dried using a hot air dryer (Bosch, BS‐6612, Germany) at 65°C for 6 h. The dried samples were ground in a herbal product grinder (Spice &Herb Grinder IC‐25B, China) and sieved to a particle size of 215 μm. Powdered samples were stored in polyethylene packages at 4°C for further use in Erişte production and compositional analysis. Rice flour, eggs, and salt were obtained from the local market in Mersin, Turkey.

### 2.2 Gluten‐Free Eriste (GFE) Production

The production of GFE was carried out by modifying the method described by Demir et al. (2021). For the GFE0 sample, the formulation included 200 g of rice flour, 30 g of whole eggs, and 1 g of salt. In the enriched GFE samples, ALP was incorporated as a rice flour substitute at levels of 5%, 10%, and 15% (*w*/*w*). The amount of water added varied between 40 and 50 mL, depending on the dough consistency (Table 1).

**FIGURE 1:**
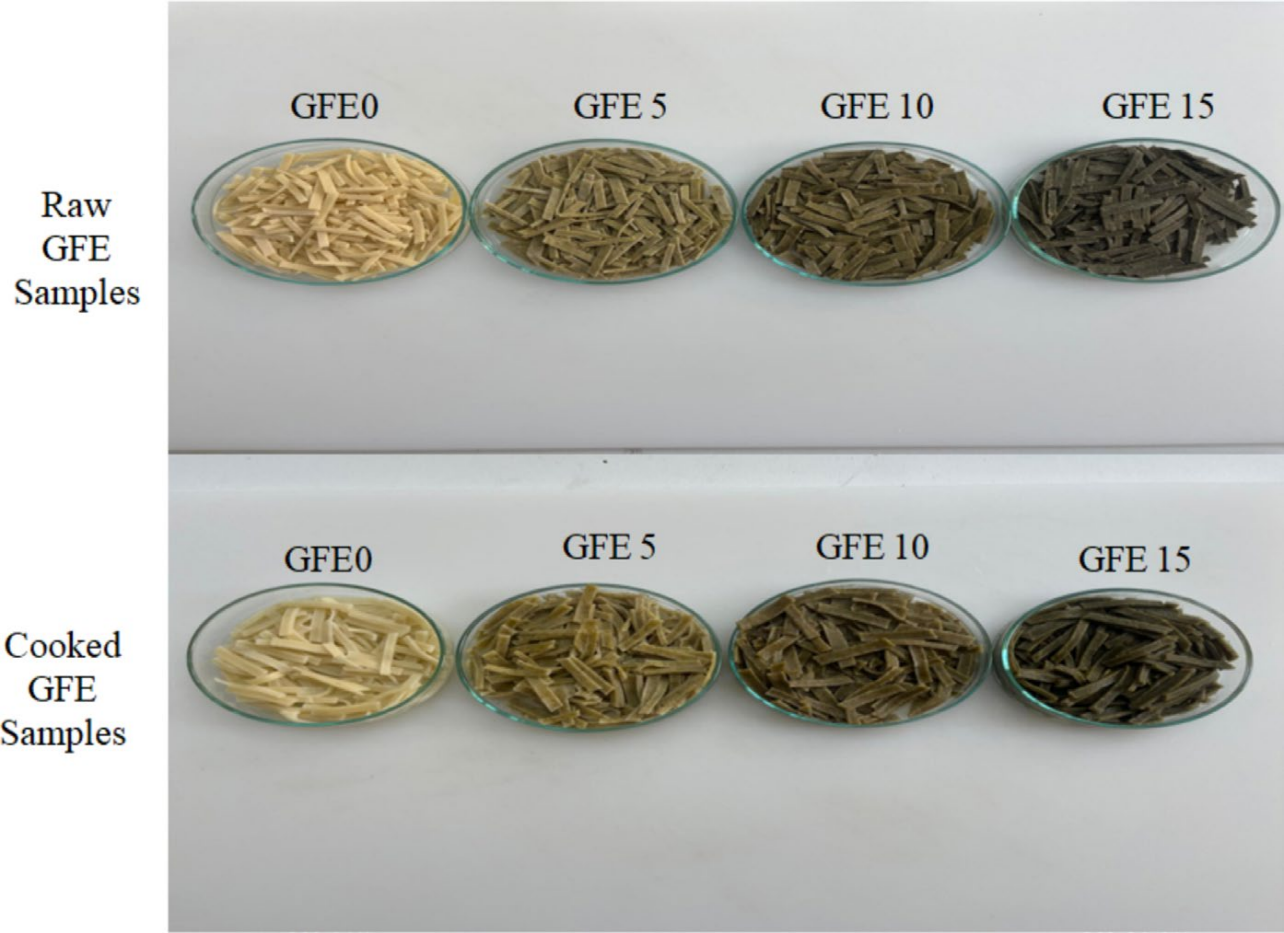
GFE samples enriched with ALP: raw and cooked forms. ALP: avocado leaf powder; GFE, gluten‐free Erişte.

All ingredients were first mixed and then kneaded for 8 min using a mixer (KitchenAid, USA). The resulting dough was rolled out manually with a rolling pin and cut into strips measuring 2 mm in thickness and 3.5 cm in width using a knife. The GFE samples were dried in an incubator at 50°C for 12 h and subsequently stored in polyethylene bags at 4°C (Figure 1).

### 2.3 Physicochemical Analysis of ALP and GFE Samples

The moisture, protein, fat, ash, and total dietary fiber contents of the ALP and GFE samples were analyzed according to the procedures outlined by AACC (2000). Results were expressed as percentages. Carbohydrate content was calculated by difference, subtracting the moisture, protein, fat, ash, and fiber residue from 100. Energy values (kcal) were calculated by applying Atwater conversion factors: 4 kcal/g for protein and carbohydrate, and 9 kcal/g for fat (Zandonadi et al. 2012). The color values of the samples were measured using a Minolta CR 400 colorimeter (Konica Minolta, Japan). The L* value ([0] black to [100] white), a* value ([+] red to [−] green), and b* value ([+] yellow to [−] blue) were evaluated based on the CIE Lab* color coordinate system established by the International Commission on Illumination. The total color difference between (Δ*E*) samples was calculated using Equation (1) (Fradinho et al. 2019).

(1) $$\Delta E=\sqrt{{\left(L^{*}-L\right)}^{2}+\left(a^{*}-a^{2}\right)+{\left(b^{*}-b\right)}^{2}}$$

To determine the total phenolic content and antioxidant activity of the ALP and GFE samples, an extraction procedure was performed. Five grams of each sample was mixed with 50 mL of 80% methanol (methanol: water, 80:20, *v*/*v*) and shaken in a water bath (Nuve BM302, Turkey) at 24°C ± 1°C. The mixture was then centrifuged (Nuve NF 800R, Turkey) at 4500 rpm for 15 min (Ozdemir et al. 2022).

For total phenolic content determination, 0.2 mL of the supernatant (extract) was transferred into a test tube, and 1.5 mL of Folin–Ciocalteu reagent (10%, *v*/*v*, in water) was added. The mixture was allowed to stand in the dark for 5 min, followed by adding 1.5 mL of sodium carbonate solution (7.5%, *w*/*v*). The mixture was incubated at room temperature for 90 min. After incubation, the absorbance of the solution was measured at 765 nm using a UV–VIS spectrophotometer (UV‐1601, Rayleigh, BFRL, China). The results were expressed as mg of gallic acid equivalents (GAE) per 100 g of sample (Ozdemir et al. 2022).

The antioxidant activity of the samples was determined using the DPPH method described by Brand‐Williams et al. (1995). For this analysis, 100 μL of extract was mixed with 3900 μL of DPPH solution (3.94 mg/100 mL in methanol) and kept in the dark for 30 min. After the incubation period, the absorbance of the solution was measured at 515 nm using a UV–VIS spectrophotometer (UV‐1601, Rayleigh, BFRL, China). The results were expressed as mg Trolox equivalents per 100 g of sample.

The contents of Fe, Mg, K, and Ca in the samples were determined using a modified method based on Levent (2017). For mineral analysis, 400 mg of the sample was digested using a microwave digestion system (Berghof Speedwave MWS‐2, Germany) with 5 mL of sulfuric acid, following the wet digestion method. The mineral content of the resulting solutions was measured using an atomic absorption spectrometer (Perkin Elmer Analyst 400, USA).

### 2.4 Cooking Properties of GFE Samples

To determine the cooking properties of GFE samples, a total of 10 g of GFE0, GFE5, GFE10, and GFE15 were boiled for 7, 9, 13, and 16 min, respectively, in a beaker containing 250 mL of distilled water without salt (Erişte‐to‐water ratio of 1:10) in a beaker. The cooking times varied depending on the disappearance of the opaque center, which marked the endpoint of cooking (AACC 2000). For the determination of cooking loss, 10 g of GFE samples were cooked in 250 mL of water in a 500 mL beaker using a water bath set to 98°C ± 2°C for 18 min. After cooking, the samples were drained, and the cooking water was collected. The water residue was then dried in an oven (Nuve, EN 400, Turkey) at 135°C. Cooking loss was calculated as a percentage based on the dried residue weight (Demir et al. 2021).

### 2.5 Determination of Resistant Starch, Non Resistant Starch, and Total Starch Content of GFE Samples

The resistant, non‐resistant, and total starch contents of GFE samples were enzymatically determined using the Megazyme Resistant Starch Assay Kit (Megazyme International Ltd., Ireland). In the analysis, samples (100 mg) were incubated in a shaking water bath (Nuve BM302, Turkey) at 37°C for 16 h with pancreatic α‐amylase and amyloglucosidase enzymes. To stop the enzymatic reaction, 4 mL of ethanol (99%, *v*/*v*) was added, followed by centrifugation at 3000 rpm for 10 min. After centrifugation, the supernatant was separated to determine the non‐resistant starch content. The remaining solid residue was washed with 8 mL of ethanol (50%, *v*/*v*) and centrifuged (Nuve NF 800R, Turkey) again (3000 rpm, 10 min). After removing the supernatant, 2 mL of 2 M potassium hydroxide solution was added to the solid residue and mixed in a water bath for 20 min. Then, 8 mL of sodium acetate buffer (pH 3.8) and 0.1 mL of amyloglucosidase enzyme were added. The solution was incubated at 50°C for 30 min and then centrifuged (Nuve NF 800R, Turkey) (3000 rpm, 10 min). For further analysis, 0.1 mL of the supernatant was mixed with 3 mL of glucose oxidase‐peroxidase‐aminoantipyrine (GOPOD) reagent and incubated at 50°C for 20 min. The absorbance of the solution was measured at 510 nm using a spectrophotometer (UV‐1601, Rayleigh, BFRL, China). Resistant starch, non‐resistant starch, and total starch contents were calculated using Equations ((2), (3), (4)) (Raungrusmee et al. 2020).

(2) $$\text{Resistant starch}\;\left(\%\right)=\left(\text{A1}\times F\right)/W\times 90$$

(3) $$\text{Non resistant starch}\;\left(\%\right)=\left(\text{A2}\times F\right)/W\times 90$$

(4) $$\text{Total starch}\;\left(\%\right)=\text{Resistant starch}+\text{Non resistant starch}$$

### 2.6 Determination of Glycemic Index (GI) of GFE Samples

The in vitro glycemic index (GI) of the GFE samples was determined based on the method proposed by Goñi et al. (1997), with slight modifications. The samples (50 mg) were homogenized with 10 mL of HCl–KCl buffer solution (pH 1.5) and then mixed with 0.2 mL of pepsin‐containing HCl–KCl buffer. The mixture was incubated in a shaking water bath at 40°C for approximately 1 h. After incubation, the sample volume was adjusted to 25 mL using Tris‐maleate buffer (pH 6.9), and the pH was adjusted to 6.9 with 1 M NaOH solution. To hydrolyze the starch in the samples, 5 mL of α‐amylase‐containing Tris‐maleate buffer was added, and the mixture was incubated in a shaking water bath at 37°C for 3 h. During incubation, 1 mL aliquots were taken at specific intervals (20, 30, 60, 90, 120, and 180 min) and immediately placed in a boiling water bath to inactivate the amylase enzyme. The samples were then stored at +4°C. At the end of the incubation period, 3 mL of 0.4 M sodium acetate buffer (pH 4.75) and 60 μL of amyloglucosidase enzyme were added to each sample, followed by incubation in a water bath at 60°C for 45 min. After incubation, 0.1 mL of the sample was mixed with 3 mL of GOPOD reagent and incubated at 45°C for 20 min, and its absorbance was measured at 510 nm using a spectrophotometer (UV‐1601, Rayleigh, BFRL, China). Starch hydrolysis curves were generated for each sample, and the area under the curve (AUC) was calculated. The hydrolysis index (HI) of each sample was determined as the ratio of its AUC to that of the reference sample (white bread) and expressed as a percentage.

(4) $$\text{Glycemic index}\;\left(\%\right)=39.71+\left(0.549\times \text{Hydrolysis Index}\right)$$

### 2.7 Textural Analysis of GFE Samples

The texture of raw and cooked GFE samples was evaluated using a two‐cycle compression test. The analysis was performed with a 50 kg load cell equipped with compression platens (P/75). The pre‐test speed was set at 1 mm/s, while the test speed was 0.5 mm/s, with a strain level of 50%. To standardize the samples, strips of cooked pasta measuring 4 cm in length were prepared. Each sample was placed on the base and subjected to two consecutive compression cycles, generating a complete compression‐relaxation‐tension profile curve. Hardness, adhesiveness, and chewiness were evaluated in cooked samples based on the force–distance curve, while hardness was exclusively assessed in the raw samples. (Martinez et al. 2007).

### 2.8 Sensory Evaluation of GFE Samples

The sensory properties of the cooked GFE samples were evaluated by a panel of 16 trained members (6 males and 10 females, aged 25–45 years), consisting of academic staff from Toros University. The Ethics Committee of Toros University in Turkey assessed and approved the research (approval number 04/71, record date: April 10, 2025). Before the evaluation, the panelists participated in a training session to familiarize themselves with the samples, evaluation criteria, and the sensory evaluation process. During the sensory analysis, each panelist received randomly coded samples in identical white plates under controlled lighting conditions. Unsalted crackers and water were provided to cleanse the palate between tastings. Each panelist assessed the samples individually in isolated booths to minimize external influences. The samples were assessed based on color, taste, hardness, stickiness, and overall preference using a 9‐point hedonic scale (1: disliked extremely, 9: liked extremely) (Singh et al. 2023).

### 2.9 Statistical Analysis

The data obtained in the study were analyzed using SPSS 20 (SPSS Inc., USA). Analysis of variance (ANOVA) was performed with a significance level set at *p* < 0.05. When significant differences were observed, multiple comparisons were made using Duncan's test to determine which treatment means differed significantly, with all tests conducted at a 95% confidence level. In addition, Pearson correlation analysis was applied to find correlation between some physicochemical and sensory properties of GFE samples. All measurements were performed in triplicate, and results are presented as mean ± standard deviation.

**TABLE 1:** GFE formulation enriched with ALP.

Ingredients	GFE0	GFE5	GFE10	GFE15
Rice flour (g)	100	95	90	85
ALP (g)	0	5	10	15
Egg (g)	30	30	30	30
Salt (g)	1.5	1.5	1.5	1.5
Water (mL)	40	40–50	40–50	40–50

Abbreviations: ALP, avocado leaf powder; GFE, gluten‐free Erişte.

## 3 Results and Discussion

### 3.1 ALP and Rice Flour Composition

The physicochemical and nutritional properties of ALP and rice flour used in GFE production are given in Table 2. The results indicate that ALP has higher ash, crude protein, crude fat, total phenolic content, antioxidant activity, and mineral composition (Fe, Mg, K, and Ca) compared to rice flour, whereas its moisture content was lower. The moisture, ash, crude protein, crude fat, and total dietary fiber contents of ALP and rice flour were determined as 9.11%, 8.38%, 22.05%, 3.09%, 19.27%, and 12.05%, 0.57%, 6.85%, 0.35%, 0.68%, respectively. Arukwe et al. (2012) highlighted in their study on the physicochemical properties of avocado fruit, leaves, and seeds that avocado leaves exhibit superior protein (25.54%), fiber (38.40%), and ash (19.38%) content. Additionally, the researchers emphasized that ALP, with its low moisture content (5.33%), has a longer shelf life. When examining the mineral composition of ALP and rice flour, ALP was found to contain significantly higher amounts of Fe, Mg, K, and Ca, being approximately 11.6, 10.2, 4.4, and 238.7 times higher than those in rice flour, respectively. Literature studies have also reported similar findings, indicating that ALP, rich in macro‐ and micro‐nutrients, is frequently used in the food industry, particularly in tea formulations (Duarte et al. 2016; Cincotta et al. 2024).

**TABLE 2:** Physicochemical and bioactive properties of ALP and rice flour.

Property	ALP	Rice flour
Moisture (%)	9.11 ± 0.06	12.05 ± 0.11
Ash (%)	8.38 ± 4.10	0.57 ± 0.04
Protein (%)	22.05 ± 2.37	6.85 ± 0.09
Crude fat (%)	3.09 ± 0.19	0.35 ± 0.03
Total fiber content (%)	19.27 ± 2.61	0.68 ± 0.04
Total phenolic content (mg GAE/100 g)	728.65 ± 37	35.22 ± 0.33
Antioxidant activity (mg Trolox/100 g)	321.37 ± 0.23	12.27 ± 0.59
Fe (mg/100 g)	19.22 ± 2.36	1.66 ± 0.05
Mg (mg/100 g)	379 ± 7.68	37.06 ± 6.33
K (mg/100 g)	488 ± 11.08	111.92 ± 7.24
Ca (mg/100 g)	1153 ± 33.17	4.83 ± 2.32
L*	45.33 ± 0.06	92.23 ± 0.06
a*	−6.91 ± 0.12	0.10 ± 0.03
b*	26.25 ± 0.16	7.41 ± 0.08

Abbreviations: ALP, avocado leaf powder; GFE, gluten‐free Erişte.

The total phenolic content of ALP (728.65 mg GAE/100 g) was approximately 21 times higher than that of rice flour. Murathan and Kaya (2020) reported that avocado leaves have a higher phenolic content than fruit pulp, with a total phenolic content of 352.30 mg GAE/100 g. Avocado leaves contain various phenolic compounds, including luteolin, rutin, quercetin, and apigenin. These compounds contribute to the health benefits of avocado leaves and their products due to their high antioxidant properties (Duarte et al. 2016). ALP has an antioxidant capacity of 321.37 mg Trolox/100 g, which is approximately 26 times higher than rice flour due to the phenolic compounds in the leaves. Furthermore, studies have also shown that the antioxidant capacity of avocado leaves was higher than that of the fruit and seed due to the bioactive compounds responsible for their antioxidant properties (Çelik et al. 2023; Arukwe et al. 2012).

As seen in Table 2, the color values of ALP were measured as L*: 45.33, a*: −6.91, and b*: 26.45, indicating a yellowish undertone in dark green shades, whereas the color values of rice flour were L*: 92.23, a*: 0.10, and b*: 7.41, reflecting a light and neutral tone. In the literature, it is widely reported that the color characteristics of plant powders are directly related to pigmentation derived from chlorophyll, carotenoids, and phenolic compounds (Nabi et al. 2023). The low L* value and negative a* value in ALP suggest a high chlorophyll concentration and other green pigments. In contrast, the high b* value indicates the presence of yellowish pigments such as carotenoids. In contrast, the high L* value and low a* and b* values of rice flour indicate a low pigment content, resulting in a white and neutral color profile. Additionally, the presence and oxidation of phenolic compounds may significantly influence the color properties of ALP.

### 3.2 Physicochemical Properties of the GFE Samples

The physicochemical analysis results of the GFE samples are given in Table 3. The moisture content of the samples ranged from 10.22% (GFE15) to 11.22% (GFE0), with a significant decrease observed as the proportion of ALP increased in the formulation (*p* < 0.05). This trend may be attributed to the lower water‐binding capacity of ALP compared to rice flour. This decrease in moisture content may enhance the microbial stability of the samples, potentially extending their shelf life (Süfer 2022). In addition, the ash and protein contents of the samples increased compared to the GFE0, which is likely attributed to the macro‐ and micro‐nutrient‐rich components such as dietary fiber, proteins, calcium, potassium, and magnesium in ALP. The crude fat content of the samples ranged from 4.09% (GFE0) to 4.16% (GFE15), and the differences were not statistically significant (*p* > 0.05). Furthermore, the total dietary fiber content increased approximately sevenfold compared to the GFE0, depending on the proportion of ALP in the GFE formulation. Given the limited fiber content of rice flour (0.68%), the addition of ALP (19.27%) enhances the fiber content of the GFE, potentially contributing to gut health, particularly for individuals following a gluten‐free diet. Jalgaonkar et al. (2018) reported that fortifying cake with 3% moringa leaf powder increased its ash content from 0.82% (GFE0) to 0.94% and its protein content from 12.34% (GFE0) to 14.44% (*p* < 0.05). Similarly, the incorporation of 5% amaranth leaf powder in cake formulations significantly increased ash, protein, and total dietary fiber contents compared to the GFE0, with increases of approximately 2.8, 1.19, and 5.75‐fold, respectively (Cárdenas‐Hernández et al. 2016). The energy values of the GFE samples were determined to range between 368.85 kcal (GFE0) and 346.08 kcal (GFE15), demonstrating a gradual decline with increasing levels of ALP in the formulation. This decrease closely corresponds with the reduction in carbohydrate content, which decreased from 71.66% in GFE0 to 61.12% in GFE15. Such a trend is in line with previous findings indicating that substituting refined gluten‐free ingredients (e.g., rice flour, as used in GFE0) with those rich in dietary fiber and phytochemicals tends to reduce the energy density of gluten‐free products (Melini and Melini 2019). Given that ALP contains fewer digestible carbohydrates and is enriched with fiber and polyphenolic compounds, its incorporation likely contributes to a lower caloric value by decreasing starch concentration and increasing the proportion of nondigestible components (Jimenez et al. 2021). From a nutritional perspective, this reduction is particularly meaningful for individuals with celiac disease and those following gluten‐free diets, which are frequently associated with high caloric density and limited nutritional variety due to the predominant use of refined flours such as rice and corn (Melini and Melini 2019). Over time, such formulations may predispose individuals to excessive energy intake, weight gain, and metabolic disturbances, particularly in gluten‐sensitive populations (Jnawali et al. 2016). In this context, the observed 6.1% decrease in energy content in the GFE15 sample could be advantageous for calorie‐conscious consumers and may support energy intake moderation without compromising product quality.

**TABLE 3:** Nutritional, bioactive, and textural properties of GFE samples.

Property	GFE0	GFE5	GFE10	GFE15
Moisture (%)	11.22 ± 0.02^d^	10.73 ± 0.03^c^	10.41 ± 0.21^b^	10.22 ± 0.03^a^
Ash (%)	0.65 ± 0.03^d^	0.85 ± 0.04^c^	1.12 ± 0.05^b^	1.33 ± 0.04^a^
Protein (%)	11.35 ± 0.46^d^	12.30 ± 0.10^c^	14.24 ± 0.20^b^	16.04 ± 0.16^a^
Crude fat (%)	4.09 ± 0.08^a^	4.12 ± 0.04^a^	4.14 ± 0.05^a^	4.16 ± 0.04^a^
Total fiber content (%)	1.03 ± 0.06^d^	5.82 ± 0.14^c^	6.18 ± 0.06^b^	7.13 ± 0.11^a^
Carbohydrate (%)	71.66 ± 0.15^a^	66.18 ± 0.19^b^	63.91 ± 0.21^c^	61.12 ± 0.14^d^
Energy (kcal)	368.85 ± 3.27^a^	351.00 ± 2.93^b^	349.86 ± 2.89^c^	346.08 ± 3.84^d^
Total phenolic content (mg GAE/100 g)	46.72 ± 0.60^d^	60.35 ± 0.35^c^	75.39 ± 0.53^b^	90.63 ± 0.46^a^
Antioxidant activity (mg Trolox/100 g)	27.78 ± 0.49^d^	35.32 ± 0.45^c^	42.29 ± 0.32^b^	50.44 ± 0.43^a^
Fe (mg/100 g)	0.80 ± 0.02^d^	2.12 ± 0.04^c^	3.47 ± 0.08^b^	4.89 ± 0.09^a^
Mg (mg/100 g)	15.46 ± 0.05^d^	19.05 ± 0.06^c^	21.37 ± 0.04^b^	23.02 ± 0.06^a^
K (mg/100 g)	65.44 ± 0.49^d^	72.23 ± 0.15^c^	85.19 ± 0.23^b^	91.12 ± 0.10^a^
Ca (mg/100 g)	19.11 ± 0.10^d^	22.33 ± 0.21^c^	28.13 ± 0.11^b^	35.16 ± 0.06^a^
L*				
Raw	53.77 ± 0.45^a^	37.16 ± 0.62^b^	29.78 ± 0.38^c^	21.78 ± 1.44^d^
Cooked	61.78 ± 0.98^a^	52.23 ± 0.36^b^	45.12 ± 0.29^c^	39.86 ± 0.28^d^
a*				
Raw	3.37 ± 0.10^a^	−1.17 ± 0.10^b^	−3.25 ± 0.04^c^	−5.33 ± 0.45^d^
Cooked	2.61 ± 0.16^a^	−2.44 ± 0.08^b^	−4.17 ± 0.04^c^	−6.00 ± 0.11^d^
b*				
Raw	35.67 ± 0.42^a^	20.64 ± 0.29^b^	16.12 ± 0.11^c^	14.19 ± 0.14^d^
Cooked	25.79 ± 0.11^a^	19.51 ± 0.28^b^	15.40 ± 0.26^c^	13.57 ± 0.32^d^
Δ*E*				
Raw	—	22.86 ± 1.23^c^	31.65 ± 1.46^b^	39.50 ± 1.27^a^
Cooked	—	12.50 ± 0.71^c^	20.77 ± 0.80^b^	26.53 ± 0.83^a^
Cooking loss (%)	10.53 ± 0.03^a^	7.82 ± 0.07^b^	7.01 ± 0.05^c^	6.47 ± 0.02^d^
Hardness (N)				
Raw	11.97 ± 0.08^d^	12.85 ± 0.12^c^	13.42 ± 0.09^b^	14.61 ± 0.11^a^
Cooked	5.21 ± 0.03^d^	5.56 ± 0.04^c^	5.82 ± 0.03^b^	6.11 ± 0.08^a^
Adhesiveness (N s)	0.88 ± 0.01^a^	0.72 ± 0.04^b^	0.65 ± 0.02^c^	0.58 ± 0.01^d^
Chewiness (N mm)	3.20 ± 0.02^d^	3.71 ± 0.05^c^	4.33 ± 0.04^b^	4.52 ± 0.03^a^

*Note:* ^a–d^Indicates that there is a significant difference between different letters on the same row (*p* < 0.05).

Abbreviation: GFE, gluten‐free Erişte.

The total phenolic content and antioxidant activity of GFE samples are given in Table 3. The enrichment with ALP resulted in a gradual increase in the bioactive properties of the samples (*p* < 0.05). The total phenolic content ranged from 46.72 mg GAE/100 g (GFE0) to 90.63 mg GAE/100 g (GFE15), while the antioxidant activity varied between 27.78 mg Trolox/100 g (GFE0) and 50.44 mg Trolox/100 g (GFE15). Notably, the GFE15 sample exhibited 1.94 times higher total phenolic content and 1.82 times more excellent antioxidant activity than the GFE0. This increase is attributed to the effective incorporation of the rich phenolic compounds (flavonoids, phenolic acids, and tannins) and antioxidants present in ALP into the food matrix. These bioactive compounds are crucial in neutralizing free radicals and reducing oxidative stress (Yamassaki et al. 2017). Weenuttranon et al. (2023) reported that including 6% mulberry leaf powder, an industrial by‐product, in pasta formulations resulted in total phenolic content and antioxidant activity approximately 16 and 11 times higher than the control samples, respectively. The researchers attributed this increase to the phenolic composition of the leaves. Similar findings have been reported by Simonato et al. (2021) for pasta enriched with moringa leaf powder (0%–15%) and by Qumbisa et al. (2021) for noodles supplemented with amaranth leaf powder (0%–3%). These studies highlight the potential of various leaf powders in enhancing the bioactive properties of the final product.

The mineral content of GFE samples is given in Table 3. The use of ALP significantly increased the Fe, Mg, K, and Ca contents of the samples (*p* < 0.05). GFE15 exhibited mineral contents that were 6.11, 1.49, 1.39, and 1.84 times higher than those of the GFE0, respectively. The daily recommended intake of minerals for adults is 15 mg of iron, 350 mg of magnesium, 3000 mg of potassium, and 1000 mg of calcium (Çelik 2021). Consumption of 100 g of GFE15 provided Fe, Mg, K, and Ca at rates of 32.6%, 3.5%, 4.56%, and 6.22% of the daily requirement, respectively. In contrast, the GFE0 contributed 5.33%, 1.9%, 3.2%, and 4.17% for these minerals, respectively. These findings suggest that ALP enrichment improves the nutritional value of GFE samples by increasing their mineral content, which may help mitigate micronutrient deficiencies, particularly iron deficiency, commonly observed in celiac patients due to malabsorption. Similar to the current study, Jalgaonkar et al. (2018) found that using 3% moringa leaf powder in pasta production increased the iron and calcium content by 41.18% and 77.57%, respectively, compared to the control samples. This finding highlights that leaf powders, which are typically considered waste despite their high nutritional composition, can serve as enriching agents in functional food production.

The color properties of raw and cooked GFE samples are given in Table 3. Adding ALP to the formulation led to significant changes in all color parameters (*p* < 0.05). In the enriched GFE samples, brightness (L*), greenness (a*), and yellowness (b*) values decreased as the usage of ALP increased. This indicates that the samples became darker, greener, and less yellow in appearance. Notably, the GFE15 sample exhibited the lowest L*, a*, and b* values, showing the most pronounced color change. This color alteration was attributed to chlorophyll pigments in avocado leaves (Murathan and Kaya 2020). Significant changes were observed in the color parameters of GFE samples after cooking. While brightness (L*) increased, a* (greenness) and b* (yellowness) values decreased. The cooking process led to the degradation of chlorophyll pigments in ALP‐enriched GFE, resulting in alterations in color pigmentation. The primary reason for this change is the conversion of chlorophyll into pheophytin during thermal processing (Gaur et al. 2006). The formation of pheophytin shifted the original green color of the samples to a dull, olive‐like tone, indicating that the pigments in avocado leaves lost stability due to heat exposure. Similar findings have been reported in the literature, with Cemin et al. (2014) observing that pasta enriched with 25% broccoli leaf powder and Weenuttranon et al. (2023) finding that pasta fortified with 6% mulberry leaf powder exhibited an increase in brightness and a decrease in a* values after cooking. These changes have been associated with starch gelatinization during cooking, which affects surface texture and light reflection (Zhang, Fan, et al. 2023).

As expected, the incorporation of (ALP) significantly altered the color of GFE samples, shifting from the light‐yellow tone of the GFE0 to darker green‐brown hues in the enriched samples, due to the presence of natural leaf pigments. The most prominent change was the color difference between raw and cooked samples. While high Δ*E* values were recorded in raw GFE samples (22.86–39.50), cooking led to a notable decrease in Δ*E* (12.50–26.53), suggesting partial pigment degradation or leaching during thermal processing. Nevertheless, the Δ*E* values in cooked GFE samples remained above the threshold of visual perception, particularly in the 10% and 15% formulations, indicating that the color shift remained noticeable even after cooking (*p* < 0.05). Similar findings were reported by Cemin et al. (2014), who enriched pasta with broccoli and spinach leaves and observed high Δ*E* values after cooking, attributing this to the heat sensitivity of chlorophyll‐rich pigments.

### 3.3 Cooking Loss of GFE Samples

The cooking loss (%) of GFE samples ranged from 6.47% to 10.53%. Cooking loss is a critical quality indicator representing the amount of dissolved starch and protein released into the cooking water. It is considered one of the most important quality parameters in pasta technology (Diamante et al. 2019). Hoseney (1986) stated that high‐quality pasta should have a cooking loss below 12%, as higher values indicate poor structural integrity and excessive starch loss during cooking. In the current study, the addition of ALP reduced cooking loss, positively impacting the quality of GFE samples. The decrease in cooking loss was correlated with the increasing proportion of ALP, yet all values remained within the acceptable threshold of 12%. This decrease is likely due to the high dietary fiber content and bioactive compounds in ALP, as shown in Table 2, which may enhance the water‐holding capacity and improve the integrity of the starch matrix, thereby minimizing free starch release during cooking. Similar findings have been reported in the literature for gluten‐free pasta enriched with different leaf powders. Studies have shown that adding spinach and beet leaf powders increases fiber content, reducing cooking loss (Smuda et al. 2024). Likewise, a decrease in cooking loss was observed in noodles enriched with moringa leaf powder as fiber content increased (Mpalanzi et al. 2023).

### 3.4 Textural Properties of GFE Samples

The textural analysis results of GFE samples are given in Table 3. The hardness of the raw and cooked samples ranged from 5.21 N (GFE0) to 6.11 N (GFE15) and from 11.97 N (GFE0) to 14.61 N (GFE15). It was observed that as the usage of ALP increased in the formulation, the hardness of the GFE also increased. This increase suggests that the dietary fiber and phenolic content of ALP strengthen the Erişte matrix, creating a more compact and hard structure (Baigts‐Allende et al. 2023). Similarly, a study by Kim and Chung (2017) reported that the hardness of cooked noodles enriched with 0%, 2%, 4%, 6%, and 8% moringa leaf powder gradually increased. The researchers attributed this to the differences in fiber and protein content in moringa leaves, stating that hardness further increased due to protein denaturation. In the raw GFE samples, the hardness values were significantly higher than in the cooked samples, which is consistent with the inherently rigid and brittle nature of dry Erişte matrices. The observed decrease in hardness after cooking aligns with previous findings (Mpalanzi et al. 2023; Süfer 2022), which associate such softening with starch gelatinization, protein denaturation, and the solubilization of phenolic and fiber components during thermal processing. Despite the absence of gluten, the increasing trend in both raw and cooked GFE samples suggests that ALP contributed to the formation of a denser and firmer structure through fiber–starch and possible polyphenol–protein interactions (Khatkar and Kaur 2018). The adhesiveness of the GFE samples ranged from 0.58 (GFE15) to 0.82 (GFE0) and decreased with the addition of ALP. This decrease in adhesiveness can be explained by the ability of ALP to bind water more effectively within the Erişte structure, preventing the release of free starch onto the surface. Similarly, a study by Mpalanzi et al. (2023) observed a significant reduction in the adhesiveness of noodles enriched with moringa leaf powder as the concentration of moringa increased. This was attributed to moringa leaves' high dietary fiber content, which enhances water retention capacity and modifies starch gelatinization, thereby reducing adhesiveness. The high fiber content of ALP used in the current study supports the consistency of our findings with those reported in the literature. The chewiness of the samples ranged from 16.82 (GFE0) to 22.45 (GFE15) and increased with the addition of ALP. This increase in chewiness is associated with the noodle becoming more elastic and resistant (Huang et al. 2024). As hardness increased, the structural integrity of the noodle was maintained, requiring more force during mastication.

### 3.5 Resistant Starch, Non‐Resistant Starch, and Glycemic Index (GI) Content of GFE Samples

The resistant starch, non‐resistant starch, and glycemic index (GI) values of GFE samples are given in Table 4. The resistant starch content of the samples ranged from 2.81% (GFE0) to 6.26% (GFE15), increasing with the use of ALP (*p* < 0.05). The rich phenolic composition of avocado leaves may enhance starch resistance to enzymatic hydrolysis by forming complexes through hydrophobic and hydrogen bonding interactions (Zhang, Wang, et al. 2023). Similarly, the literature indicates that polyphenol‐containing compounds promote resistant starch formation (Gutiérrez and Tovar 2021). Avocado leaves may slow down starch digestion, thereby increasing resistant starch content.

**TABLE 4:** Resistant starch, non‐resistant starch, total starch, and GI values of GFE samples.

Sample	Resistant starch (%)	Non‐resistant starch (%)	Total starch (%)	GI (%)
GFE0	2.81 ± 0.29^d^	71.78 ± 1.57^a^	74.59 ± 1.17^a^	82.22 ± 1.44^a^
GFE5	4.14 ± 0.30^c^	67.17 ± 1.86^b^	71.31 ± 1.09^b^	77.63 ± 1.32^b^
GFE10	5.83 ± 0.44^b^	61.37 ± 1.21^c^	68.20 ± 1.22^c^	73.43 ± 1.52^c^
GFE15	6.26 ± 0.52^a^	58.91 ± 1.18^d^	65.17 ± 1.30^d^	68.85 ± 1.63^d^

*Note:* ^a–d^Indicates that there is a significant difference between different letters on the same column (*p* < 0.05).

Abbreviations: GFE, gluten‐free Erişte; GI, glycemic index.

The high dietary fiber content may also restrict starch granule water absorption and gelatinization, contributing to resistant starch formation (Brasil et al. 2011). As resistant starch increased, a decrease in digestible starch (non‐resistant starch) content was observed (*p* < 0.05). Since digestible starch is calculated as the difference between total starch and resistant starch, an increase in resistant starch naturally led to a decrease in non‐resistant starch content. This suggests that starch becomes less digestible, potentially slowing the energy release rate. The total starch content of the samples ranged from 74.59% (GFE0) to 65.17% (GFE15), with a significant decrease observed as the usage of ALP increased (*p* < 0.05). This can be explained by avocado leaves having a low starch content and being rich in dietary fiber. Similarly, Lin (2022) reported that plant‐based dietary fiber additives dilute the total starch content in flour‐based products and alter the composition of complex carbohydrates.

As seen in Table 4, the GI of GFE ranged from 73.43% (GFE15) to 82.22% (GFE0), and it was found to decrease as the amount of ALP in the formulation increased. This effect is associated with the ability of polyphenols to inhibit starch‐digesting enzymes such as α‐amylase and α‐glucosidase (He et al. 2021). Similarly, the GI‐lowering effect of polyphenols has been observed in matcha‐enriched rice noodles (Li et al. 2023). The phenolic compounds in avocado leaves may have delayed starch digestion by inhibiting its breakdown by amylase, slowing the digestion rate. Additionally, since avocado leaves are rich in dietary fiber, they may slow digestion and delay glucose absorption (Fabek et al. 2014). This effect is particularly beneficial for reducing the GI of rice flour‐based products. The GI measures how quickly blood sugar levels rise after consuming a meal and generally categorizes foods into three groups: high GI (> 70), medium GI (56–69), and low GI (≤ 55) (Owheruo et al. 2023). Therefore, the addition of 15% ALP has the potential to transform GFE from a high‐GI food (82.22) to a medium‐GI food (68.85). This further demonstrates that incorporating ALP is a promising approach to enhancing the nutritional and health quality of rice—Erişte.

### 3.6 Sensory Evaluation of GFE Samples

The sensory evaluation results of the GFB samples are given in Table 5. In terms of color, the highest score was observed in the GF15 sample (8.00), while the lowest was recorded in the GFE0 sample (6.50). The flavonoids and chlorophyll derivatives in avocado leaves may have imparted a greenish tone to the gluten‐free noodles, enhancing their visual appeal to panelists (Gümüştepe et al. 2022). Similarly, Kim and Chung (2017) reported that noodles enriched with moringa leaf powder received higher color scores than the control, attributing this to the various functional compounds in moringa leaves. For taste evaluation, the highest score was measured in the GFE0 sample (7.83), whereas the lowest was observed in the GF15 sample (5.38). As the usage of ALP increased, taste scores decreased, which could be attributed to the bitter and astringent flavor profile caused by phenolic compounds such as quercetin, kaempferol, tannins, and anthraquinone derivatives present in avocado leaves (Gümüştepe et al. 2022). In the hardness evaluation, the highest value was recorded for GFE15 (6.23), while the lowest was found in the GFE0 sample (4.77). The decrease in hardness may be associated with the reduction in the starch content of rice flour and the effect of ALP fiber on the dough matrix. Similarly, previous studies have reported that ingredients rich in fiber and phenolic compounds tend to lower hardness scores in pasta‐like products (Sajid Mushtaq et al. 2023).

**TABLE 5:** Sensory properties of GFE samples.

Sample	Color	Taste	Hardness	Stickiness	Overall preference
GFE0	6.59 ± 1.28^d^	7.83 ± 1.70^a^	4.77 ± 0.12^d^	6.40 ± 0.80^a^	7.33 ± 1.15^d^
GFE5	7.21 ± 1.67^c^	6.50 ± 1.49^b^	5.30 ± 0.74^c^	6.47 ± 1.05^a^	7.80 ± 1.76^b^
GFE10	7.58 ± 1.93^b^	6.14 ± 1.29^c^	5.52 ± 0.98^b^	6.53 ± 1.01^a^	7.97 ± 1.18^a^
GFE15	8.00 ± 2.13^a^	5.38 ± 1.65^d^	6.23 ± 0.59^a^	6.67 ± 0.96^a^	7.56 ± 1.13^c^

*Note:* ^a–d^Indicates that there is a significant difference between different letters on the same column (*p* < 0.05).

Abbreviation: GFE, gluten‐free Erişte.

Regarding stickiness, the highest score was found in the GFE0 (6.40), while the lowest was in GFE15 (6.47), with no significant difference observed between the results (*p* > 0.05). When evaluating the overall preference of GFE samples, the GFE10 formulation received the highest score from panelists, indicating it was the most well‐accepted variant. However, as the usage of ALP exceeded 10%, a noticeable decline in overall preference was observed. Similar to the decrease in taste scores, this decline is likely attributed to the presence of phenolic compounds, which may have contributed to a more pronounced bitter and astringent flavor.

### 3.7 Correlation Between Physicochemical Parameters and Sensory Properties of GFE Samples

Pearson correlation analysis was performed to evaluate the association between selected physicochemical parameters and sensory properties of GFE samples. The complete correlation matrix is presented in Table 6. Several statistically significant correlations (*p* < 0.05) were observed, providing insights into how compositional and structural properties of GFE samples influence sensory perception. Crude fat content showed a perfect positive correlation with sensory color (*r* = 1.000, *p* < 0.05), suggesting that higher fat levels in GFE samples may enhance visual appeal, possibly due to improved glossiness or color intensity. Similarly, color was positively correlated with protein (*r* = 0.968), total phenolic content (*r* = 0.990), antioxidant activity (*r* = 0.993), hardness (*r* = 0.998), and chewiness (*r* = 0.982), indicating that nutrient‐dense and structurally firmer GFE samples tend to be perceived as more visually attractive. In contrast, color was negatively correlated with moisture (*r* = −0.993) and adhesiveness (*r* = −0.994), implying that softer and more adhesive texture samples may reduce visual appeal. Taste was negatively associated with crude fat (*r* = −0.989), total phenolic content (r = −0.966), and antioxidant activity (*r* = −0.972), suggesting that while these components enhance functionality in GFE formulations, they may contribute to bitterness or astringency, thereby reducing palatability. On the other hand, a strong positive correlation was observed between taste and adhesiveness (*r* = 0.996), potentially reflecting a greater retention of flavor in more adhesive matrices. Instrumental hardness was strongly correlated with sensory hardness (*r* = 0.983), demonstrating good agreement between instrumental and panel assessments. Additionally, protein, phenolic compounds, and antioxidant activity were all positively correlated with perceived hardness, indicating that bioactive enrichment contributes to firmer texture perception in these GFE formulations. Stickiness was positively correlated with protein (*r* = 0.984), crude fat (*r* = 0.961), and antioxidant‐related parameters, suggesting that these components may enhance perceived stickiness, possibly due to changes in the matrix structure or surface characteristics of GFE samples. Although no statistically significant correlations were detected between physicochemical parameters and overall preference, fiber content (*r* = 0.689) and chewiness (*r* = 0.544) showed moderate positive trends, suggesting that textural satisfaction may influence overall acceptance. In summary, the data indicate that in GFE samples, key physicochemical properties, especially protein, fat, fiber, and antioxidant‐related components, significantly affect sensory attributes such as color, taste, texture, and acceptability. These correlations highlight the importance of balancing nutritional enhancement with sensory quality in product development.

**TABLE 6:** Pearson correlation between some physicochemical parameters and sensory properties of GFE samples.

	Color	Taste	Hardness	Stickiness	Overall preference
Moisture (%)	−0.993**	0.986*	−0.947	−0.926	−0.568
Protein (%)	0.968*	−0.932	0.971*	0.984*	0.296
Crude fat (%)	1.000***	−0.989*	0.975*	0.961	0.477
Total fiber content (%)	0.931	−0.964*	0.872	0.817	0.689
Total phenolic content	0.990*	−0.966*	0.982*	0.982*	0.376
Antioxidant activity	0.993**	−0.972*	0.987*	0.984*	0.377
Hardness (N)	0.998	−0.984*	0.983*	0.973*	0.432
Adhesiveness (N.s)	−0.994**	0.996**	−0.955*	−0.928	−0.559
Chewiness (N.mm)	0.982*	−0.958*	0.930	0.922	0.544

* *p* < 0.05.

** *p* < 0.01.

*** *p* < 0.001.

## 4 Conclusion

In this study, the usage of ALP, a food waste by‐product, in GFE production and its effects on the nutritional, textural, and sensory properties of the product were investigated. Incorporating ALP into the formulation successfully improved these properties compared to the GFE0 sample (100% rice flour). As the ALP usage increased, notable enhancements were observed in ash, protein, fat, total dietary fiber, total phenolic content, antioxidant activity, and mineral levels, highlighting the potential of ALP to nutritionally enrich GFE samples. Textural improvements, such as increased hardness and chewiness and decreased adhesiveness, indicate that ALP contributes to a firmer and more cohesive GFE structure. Moreover, the observed reduction in total starch content and glycemic index, alongside an increase in resistant starch, suggests that ALP inclusion may support better glycemic GFE0 and improve the overall health profile of GFE samples. These findings imply that incorporating ALP not only enhances the nutritional and functional quality of GFE samples but also contributes to the development of products with added health benefits, particularly relevant for individuals with gluten sensitivity or metabolic concerns. Sensory analysis results indicated that the GFE10 sample had the highest overall preference score compared to the GFE0. These findings suggest that ALP can be utilized as a functional food ingredient and an effective additive in producing gluten‐free foods with a lower glycemic index.

## Author Contributions

**Başak Öncel:** data curation (equal), investigation (equal), methodology (equal), writing – original draft (equal), writing – review and editing (equal). **Nesibe Eryilmaz:** data curation (equal), investigation (equal), methodology (equal), writing – original draft (equal), writing – review and editing (equal).

## Conflicts of Interest

The authors declare no conflicts of interest.

## Data Availability

The data produced in this study are accessible through the corresponding author upon a request.
